# Irritable bowel syndrome and microbiome; Switching from conventional diagnosis and therapies to personalized interventions

**DOI:** 10.1186/s12967-022-03365-z

**Published:** 2022-04-11

**Authors:** Pouyan Ghaffari, Saeed Shoaie, Lars K. Nielsen

**Affiliations:** 1 Novo Nordisk Foundation Center for Biosustainability, Technical University of Denmark, 2970 Hørsholm, Denmark; 2 Centre for Host-Microbiome Interactions, Faculty of Dentistry, Oral & Craniofacial Sciences, King’s College London, London, SE1 9RT UK; 3 Science for Life Laboratory, KTH - Royal Institute of Technology, 171 21, Stockholm, Sweden; 4 Australian Institute for Bioengineering and Nanotechnology (AIBN), The University of Queensland, St. Lucia, 4072 Australia

## Abstract

The human microbiome has been linked to several diseases. Gastrointestinal diseases are still one of the most prominent area of study in host-microbiome interactions however the underlying microbial mechanisms in these disorders are not fully established. Irritable bowel syndrome (IBS) remains as one of the prominent disorders with significant changes in the gut microbiome composition and without definitive treatment. IBS has a severe impact on socio-economic and patient’s lifestyle. The association studies between the IBS and microbiome have shed a light on relevance of microbial composition, and hence microbiome-based trials were designed. However, there are no clear evidence of potential treatment for IBS. This review summarizes the epidemiology and socioeconomic impact of IBS and then focus on microbiome observational and clinical trials. At the end, we propose a new perspective on using data-driven approach and applying computational modelling and machine learning to design microbiome-aware personalized treatment for IBS.

## Introduction

Irritable bowel syndrome (IBS) is one of the most common functional gastrointestinal disorder characterized by symptoms such as chronic recurrent abdominal pain, changes in stool consistency and frequency, changes in bowel habits, flatulence and bloating. IBS is currently diagnosed by symptomatic criteria, namely the Rome IV criteria, and sensitive and specific diagnostic markers are not established yet. According to the Rome IV criteria and based on predominant stool pattern, IBS patients are stratified into four main subtypes: IBS with diarrhea (IBS-D), IBS with constipation (IBS-C), IBS with mixed bowel habits (IBS-M), and unclassified IBS [1, 2].

IBS is believed to be a multifactorial and heterogeneous condition and its pathophysiology is not completely understood. Potential factors include genetic background, gut microbiome dysbiosis, dietary habits, psychological factors, and gastrointestinal infection [3, 4]. IBS shows a clear association with other gastrointestinal disorders, chronic pain disorders such as pelvic pain and fibromyalgia, and with psychiatric conditions such as depression, anxiety, and migraine [5, 6]. Patients with IBS and inflammatory bowel disease (IBD) may show similar symptoms, but while the pathogenesis of IBD involves mucosal inflammation, the pathogenesis of IBS is not clearly understood, and there is no causative biochemical or anatomical irregularity that can be used to diagnose IBS [7]. Despite great variety of therapeutic options, there has been no standard guideline or robust therapy for IBS, leading to suboptimal treatment satisfaction for both doctors and patients [8, 9]. Currently, there is no definitive cure for IBS, and relief of symptoms is what can be achieved by treatments.

## Epidemics/Global and regional Prevalence

In 2012, a systemic review and meta-analysis covering 90 epidemiological studies across 33 countries worldwide reported a pooled global prevalence of IBS of 11.2% (95% CI: 9.8–12.8), varying widely from lowest ratio of 1.1% to highest rate of 45% between countries [10]. The origin of this variation is not clear. It may be mediated by factors such as diet, ethnicity and public health system, or might be resulted from methodological variations between studies. Gathering prevalence information of IBS subtypes is not straight forward as they show considerable overlap of symptoms and may switch over time. A couple of population studies in countries with pooled IBS prevalence of 10%, revealed IBS-D and IBS-C each contributes for approximately 30% of the diagnosed population [11, 12]. The IBS annual occurrence of new cases (IBS annual incidence rate) has not been reported for many countries, but a long-term population survey in the US shows an estimate in the range of 1–2% [13]. Worldwide analysis of IBS prevalence across 56 countries reported higher incidence rate in women than men (OR 1.67, 95% CI 1.53–1.82) [14].

## Socio-economic impact and burden

IBS has substantial negative impact on patients’ personal and work life, and consequently on their family and society. Several health-related studies revealed consistent reduction in quality of life (QOL) of IBS patients in European and North American populations [15, 16]. Reluctance to leave home and avoidance of social places was reported mainly in IBS-D patients, whereas difficulty in concentration and avoiding sex was more likely to be seen in IBS-C patients. IBS also has a negative effect on work life including less tendency to travel, reduced socializing and loss of earning. Overall, individuals with IBS report unpredictability of their symptoms and highlight that they can feel stigmatized by family and friends, who might struggle to understand the impact of IBS on their quality of life [17, 18]. In fact, patients with severe symptoms show more tendency to accept high level of risk to resolve their symptoms. For example, a questionnaire study reported that people with severe IBS are willing to give up an average of 15 years (up to 25%) of their remaining life expectancy to live free of symptoms [19].

Recently, Shorey et. al. published a qualitative systematic review about IBS patient’s perspectives on healthcare, daily living, and self-care management. They analyzed 17 studies including 299 adults that diagnosed with IBS, aged between 19 and 88 years and majority from Europe and North America. They identified four themes: (1) physical, psychological, and social impacts; (2) effects on work life; (3) handling IBS; and (4) relevant support with required sources. They also mentioned importance of the integrating technology to design IBS-related support systems to enhance patients' health literacy, countering societal stigma against IBS, and evaluating the effectiveness of the social networks to support adults living with IBS [20].

**Fig. 1:**
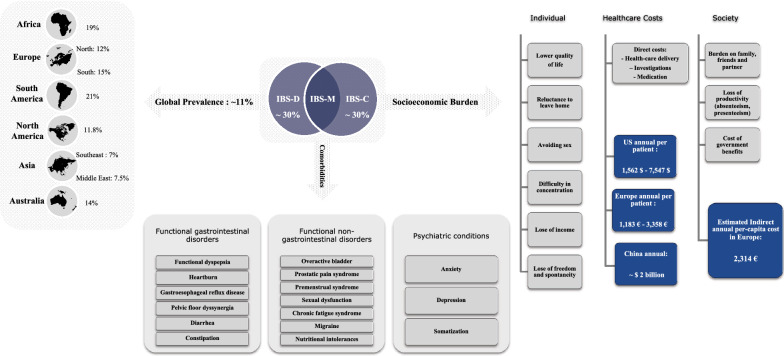
Irritable Bowel Syndrome (IBS). Global and regional prevalence [10], socio-economic burden [24] and comorbidity [6]

IBS brings substantial direct and indirect costs to patients and society. In 2013, a systemic review of the economic burden of IBS analyzing 35 studies, estimated a considerable health care cost in USA, ranging from $1562 to $ 7547 per patient per year [21]. Similarly, analysis covering data from six European countries estimated per capita cost of 1183 € to 3358 € [22]. Also, economic impact of IBS on the health care system has been estimated to be up to $ 2 billion per annum in China [23]. IBS has considerable indirect costs like other chronic conditions such as migraine and asthma, due to loss of work performance and productivity. A study encompassing data from 13 European countries estimated annual indirect cost of 2314 € per-capita for IBS (Fig. 1) [22].

## Pathophysiology and risk factors

The pathophysiology of IBS is largely undetermined and current understanding of the potential underlying mechanisms is incomplete. However, cumulative knowledge and growing evidence during the past decades suggest contribution of the gut microbiota, bile acids, food antigens and the intestinal epithelial barrier in producing anomalous responses in the main regulators of the sensory-motor functions in IBS, including immune system, the enteric nervous system (ENS), the hypothalamus–pituitary–adrenal (HPA) axis and the gut-brain axis [6, 24–26]. In addition, psychosocial factors such as stress, that influences physiological functions of the gut, and factors such as anxiety and depression, which are known to be influenced by gastrointestinal symptoms, have been acknowledged in pathophysiology of the IBS. Some investigators have reported familial aggregation of IBS and findings from twin studies have shown higher concordance in monozygotic twins compared with dizygotic twins, suggesting potential underlying genetic factors in IBS [27–29]. A recently published genome-wide association study including 53,400 people with IBS and 433,201 controls, identified six independent genetic susceptibility loci for IBS at genome-wide significance (P < 5 × 10 − 8) and all six loci were replicated at Bonferroni significance (P < 0.0083) using data from an independent panel from 23andMe (205,252 cases and 1,384,055 controls). This study reported strong genome-wide association between IBS and mood and anxiety disorders rooted to shared pathogenic pathways [30]. Due to the important role of serotonin in the brain–gut axis, genetics of serotonergic pathways, especially the serotonin transporter (SERT), have gained a great amount of attention in recent years [31]. In a meta-analysis covering more than 7,000 participants across 27 studies, authors reported significant association between SERT insertion or deletion polymorphism and the risk of IBS [32]. Female gender is a well-documented risk factor for IBS, with an average odds ratio of 1.67 across population-based studies [14]. A genome-wide association study comparing data from 9,576 IBS patients and 336,449 healthy controls in UK biobank, identified an association between IBS risk in women only and variants at a locus on chromosome 9, which might support the rationale for studying the role of sex hormones in the pathophysiology of the functional gastrointestinal disorders [33]. The corticotropin-releasing hormone (CRH) is vital to the body’s stress response and studies in Japanese subjects have identified association between single nucleotide polymorphism in genes encoding CRH receptors 1&2 and IBS symptoms, indicating possible role of the CRH pathway in IBS pathophysiology [34, 35]. Several studies have shown an association between previous bacterial or viral gastrointestinal infections and risk of developing post-infectious IBS (PI-IBS) [36, 37]. A range of bacterial pathogens have been implicated in PI-IBS, including *Clostridioides difficile1* [38], *Vibrio cholerae* [39], *Campylobacter jejuni*, *Escherichia coli* and *Salmonella enterica serovar Typhimurium* [40].

**Fig. 2:**
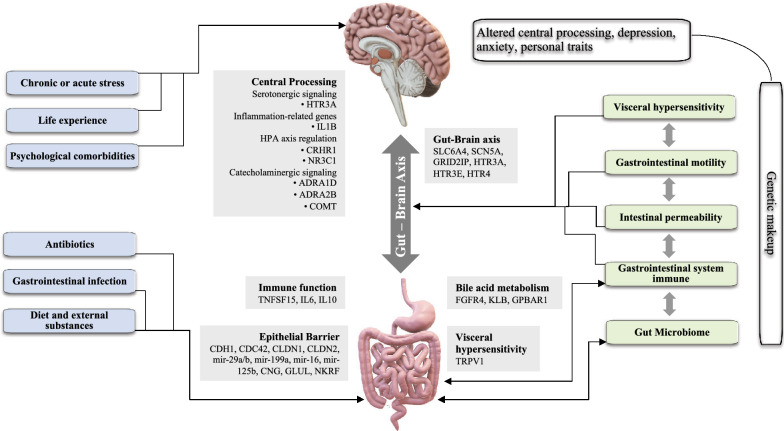
Potential interconnected factors that regulate the manifestation of IBS symptoms. IBS has a multifactorial pathophysiology and multiple interrelated pathways can influence the manifestation of symptoms. External factors are dominant, but internal factors such as gut microbiome, gastrointestinal immune system and genetic makeup is also likely to be crucial for the development and progression of symptoms. Here we summarized potential external and internal factors and genetic findings linked to underlying pathophysiological mechanisms of IBS [6, 24, 26, 30, 96]. HPA, hypothalamic–pituitary–adrenal axis; ADRA, adrenoceptor-α; aINS, anterior insula; CDC42, cell division cycle 42; CDH1, cadherin 1; CGN, cingulin; CLDN, claudin; COMT, catechol-O-methyltransferase; CRHR1, corticotropin-releasing hormone receptor 1; FGFR4, fibroblast growth factor receptor 4; GLUL, glutamate-ammonia ligase; GPBAR1, G protein-coupled bile acid receptor 1; GRID2IP, GRID2-interacting protein; HTR, 5-hydroxytryptamine receptor; IL, interleukin; KLB, Klotho-β; mir, microRNA; NKRF, nuclear factor-κB-repressing factor; SCN5A, sodium voltage-gated channel α-subunit 5; SLC6A4, solute carrier family 6 member 4; TNF, tumour necrosis factor; TNFSF15, TNF superfamily member 15; TRPV1, transient receptor potential cation channel subfamily V member 1;; NCAM1, Neural Cell Adhesion Molecule 1; CADM2, Cell Adhesion Molecule 2; PHF2, PHD Finger Protein 2; DOCK9, Dedicator Of Cytokinesis 9

Some dietary compounds might be involved in the development and progress of IBS symptoms. More than 25 years ago, it was reported that the consumption of large amounts of insoluble fiber intensifies symptoms in IBS patients [41]. Some subgroups of IBS patients experience exacerbated symptoms when consuming food containing fermentable oligosaccharides, monosaccharides, disaccharides and polyols [42]. Removal of gluten from the diet has a positive effect on a proportion of IBS patients by improving symptoms [43]. There is evidence for a role of disordered bile acid metabolism in IBS pathophysiology. Cross-sectional surveys by 23-seleno 25-homotaurocholic acid retention scanning revealed that approximately 20% of IBS patients with diarrhea show indication of idiopathic bile acid diarrhea. Also, an investigation of the association between fecal bile acids and IBS symptoms, revealed that total fecal bile acids concentration was lower in IBC-C and higher in IBC-D subtypes [6, 44]. Figure 2 summarizes risk factors, pathophysiological mechanisms, and genetics findings associated with IBS.

## IBS and Microbiome

**Fig. 3:**
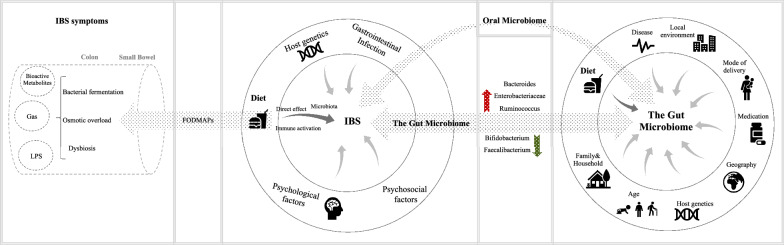
IBS-Microbiome-Diet axis. The gut microbiome might be an important factor with higher degrees of dysbiosis and altered abundance of some species observed in IBS patients. Diet might have a substantial effect on IBS symptoms through mechanisms, such as changing gut microbiota, direct effect of food, and immune activation. Fermentable oligosaccharides, disaccharides, monosaccharides and polyols (FODMAPs) might cause IBS symptoms via microbiome dysbiosis, bacterial fermentation and osmotic overload [97]. Gut microbiota composition and function is shaped by several factors from which, diet might be the key determinant of the microbiota configuration. Oral microbiome may have a potential in diagnosis and patient stratification in IBS. LPS, lipopolysaccharide

A growing body of evidence indicates that the gut microbiota plays an important role in gastrointestinal (GI) disease including IBS. The fecal microbiota of IBS patients differs significantly from healthy subjects, with potential contribution to altered bowel habits and influencing colonic transit [45, 46]. Several studies have indicated that the abundance of Bifidobacterium, Lactobacillus and Faecalibacterium is reduced, while the abundance of Veillonella, Ruminococcus and proinflammatory bacterial species such as Enterobacteriaceae is increased [47–51]. Conversely, a recent systematic review of gut microbiota in patient with IBS reported increased abundance of family Lactobacillaceae and genus Bacteroides [52]. Both higher and lower ratio of Firmicutes/Bacteroidetes, that is a rough indicator of altered microbial population, has been reported in IBS subjects [53, 54]. Reduced diversity of gut microbiome and presence of *Clostridiales*, *Prevotella* and methanogenic species has been proposed as an IBS-specific microbiome signature that associate with severity of symptoms [55]. However, this microbial signature cannot yet be explicitly correlated nor explained by application of medicines, differences in dietary habits or genetic factors. The gut metabolome, intestinal permeability and inflammatory pathways have also been suggested to play a role microbiome-related background of gastrointestinal disease [56] (Fig. 3).

These findings indicate potential influence of the gut microbiota in the development of effective treatments for IBS. To better understand the role of the gut microbiome in the IBS pathology, it is important to explore the inter-species and host-microbe interactions, as well as the interplay between microbiome composition and factors that influence the IBS severity, such as sex and psychiatric comorbidities.

## Therapeutic Interventions

The purpose of most current therapeutic interventions is to reduce visceral pain and/or to change predominant problematic bowel habits in IBS. However, an emerging field is manipulation of the gut microbiota. Table 1 provides a summary of the current approved medications for treatment of IBS-related symptoms.

**Table 1:** Summary of treatments for IBS-related symptoms [98–115]

Therapy type/class	IBS-related symptoms	Data Quality	Mechanism of action	Adverse events	References
5-HT4 receptor agonists	Constipation	High	Stimulate colonic motility and transit	Diarrhea, cramping, and cardiovascular side effects	[97, 98]
Tenapanor	Constipation	Moderate	NHE3 inhibitor, stimulates sodium + , water secretion	Diarrhea more common with active therapy	[99]
IBAT inhibitor	Constipation	Moderate	Increases colonic bile acid levels to induce secretion and motility	Diarrhea, cramping	[100]
Linaclotide	Constipation	High	Guanylate cyclase C activator, stimulate chlorine − and water secretion via CFTR; visceral analgesia	Diarrhea more common with active therapy	[97]
Plecanatide	Constipation	High		Diarrhea more common with active therapy	[101, 102]
PEG 3350	Constipation	Moderate	Osmotic secretion	Diarrhea and abdominal pain	[103]
Lubiprostone	Constipation	Moderate	Chloride channel activation and with CFTR stimulate chlorine − secretion; inhibitor of NHE3	Nausea more common with active therapy	[104]
Bile acid sequestrants	Diarrhea	Low	Bind intraluminal bile acids	Limited data	[105, 106]
5-HT3 receptor antagonists	Diarrhea	High	Retard colonic transit and reduce visceral pain	Serious adverse events with alosetron included ischemic colitis and severe constipation	[107, 108]
Rifaximin	Diarrhea	Moderate	Nonabsorbable antibiotic	Nausea more common with active therapy	[107, 109]
Eluxadoline	Diarrhea	High	κ-Opioid and μ-opioid receptor agonists and δ-opioid receptor antagonist	Serious adverse events included acute pancreatitis and sphincter of Oddi spasm	[107]
Peppermint oil	Pain	Moderate	Blocks L-type calcium ion channels on muscle, activate TRPM8 receptors on nociceptive afferents	No increase in adverse events in randomized clinical trials	[110, 111]
Antidepressants	Pain	Moderate	Psychological, antinociceptive, slow (TCA) or fast (SSRI) transit effects	dry mouth and drowsiness	[110, 112]
Antispasmodic drugs	Pain	Low	Inhibition of muscarinic Ach receptors or block calcium ion channels, GI smooth muscle	dry mouth, dizziness, and blurred vision	[113, 114]

Pain in IBS is partially the result of the smooth muscle spasm, and antispasmodic drugs such as neurokinin-type 2 receptor antagonists and calcium channel blockers are used as the first-line treatment in pain-predominant IBS patients. IBS is associated with psychological disorders and low-dose antidepressants, such as selective serotonin reuptake inhibitors (SSRIs) and tricyclic antidepressants (TCAs), are recommended for the treatment of pain in patients. Simple laxatives, such as docusate and senna, are often effective first line therapy in patients diagnosed with IBS-C, followed by liaclotide as second line therapy. Antidiarrhoeals, such as μ‑opioid receptor agonist loperamide, are used to prolong the gastrointestinal transit time and to improve diarrhea in patients with IBS-D [6, 57].

Although it is not clear whether alterations in the gut microbiota in IBS patients precede or are an outcome of the disrupted local gut microenvironment condition, modulation of gut microbiota for treatment of the IBS has sparked interest in recent years. Several facts support this tendency: some pre-/probiotics can relieve IBS symptoms [58]; visceral colonic hypersensitivity that is a critical feature of the IBS can be transferred to germ-free mice by fecal transplantation [59]; randomized controlled trails with rifaximin, a no-absorbable antibiotic, revealed benefit for IBS patients [60]; a systematic review and meta-analysis including 45 studies and 21,421 individuals with infectious enteritis, has reported fourfold higher risk of developing IBS in individuals with gastrointestinal infection [61]; dietary interventions that are known to modulate gut microbiota, such as a diet low in FODMAPs (Fermentable Oligo-, Di-, Mono-saccharides And Polyols), have also reported to reduce symptoms of IBS in several randomized placebo-controlled trials [62–67]; recent observations suggest a positive effect of fecal microbiota transplantation in alleviating IBS symptoms [68, 69]. Overall, there is growing evidence supporting microbiome-based therapeutic approaches for the treatment of IBS. Table 2 summarizes a couple of recent systematic reviews and meta-analysis that evaluated the effect of probiotics, prebiotics, dietary intervention, non-absorbable antibiotics, and FMTs for the treatment of IBS.

**Table 2:**
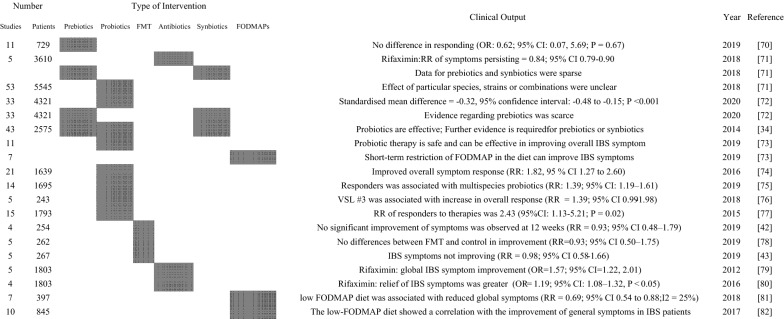
Systematic reviews with meta-analysis reporting efficacy of microbiome-based therapeutic interventions in IBS

## Host-microbiome and diet interactions

Employing personalized nutrition to modulate host-microbiota interactions is a new therapeutic avenue for disease prevention and control. The role of host genetics in shaping the composition of gut microbiota has been reported in some studies, but environmental factors overweighed the genetics [70–72]. While early life events have significant impact on the gut microbiota, it does maintain some degree of elasticity and can be shaped by later environmental factors, such as diet, hygiene, antibiotic and non-antibiotic drugs, weather, pollution, and so forth. Of these, diet is the key driving force that modulate abundance and functions of microbial species, in a fast, personalized, and reproducible manner [73–75]. Collectively, interdependent function of the three biologically and chemically interconnected systems defines an individual’s response to dietary interventions: host physiology and metabolism; the microbiota structure and state; and diet composition and timing [76, 77].

Variations in dietary macronutrients, including carbohydrates, protein and fat, significantly changes gut microbiota. Carbohydrates, dependent on their types and amounts, have complex effect on the gut microbiota. It has been reported that long-term consumption of complex carbohydrates promotes Prevotella. Dietary fibers influences microbiome ecology and elevates abundance of Bacteroidetes [78, 79]. Some bacteria can grow on specific types of carbohydrates, and consequently diet can eliminate or select for certain species. For example, grain-reduced diet can decrease abundance of Bifidobacteria that selectively degrade arabinoxylans in grains [80]. Despite common signature of response to carbohydrates within population, highly personalized shifts have been reported in response to dietary fibers, carbohydrate containing prebiotics and resistant starches [81–84]. In humans, short-term administration of diets rich in animal-protein resulted in decreased abundance of saccharolytic species (including *Ruminococcus bromii*, *Eubacterium rectale* and *Roseburia spp.*), while increased abundance of bile-tolerant species, such as Bacteroides, Bilophila and Alistipes [74]. Moreover, consumption of diet rich in plant protein resulted in elevated production of short-chain fatty acid (SCFA) and considerable increased abundance of commensals Bifidobacteria and Lactobacilli [85]. Also, Long term consumption of the animal protein diet has been associated with the Bacteroides [78] (Fig. 3).

## Exponential increase of microbiome data and need for predictive models

Big biological datasets contain the raw data required to gain insights into complex biological systems, but high-level analysis is needed to realize the potential of these data. Machine learning is a discipline in computational science where computers are trained to learn patterns from data. Machine learning methods aim to recognize patterns and to develop predictive models based on statistical associations between features from a given dataset. The machine learning algorithm typically consist of the measurement across a set of samples which are called features, and the labels that model aims to predict using features. The learning process, that is based on a set of mathematical assumptions and rules, refers to finding the optimal set of parameters that translate the features in the training dataset into correct predictions of the labels in the test dataset. In life science, features can cover one or more types of data, such as a genomic sequence, gene expression profiles, protein expression levels, protein–protein interactions, metabolite concentrations, abundance profiles or copy number alterations. Labels can be binary e.g. pathogenic or non-pathogenic, continuous e.g. growth rate, or categorical e.g., stage of disease [86, 87]. Machine learning methods can be split into two main categories: unsupervised and supervised learning. Supervised approaches are used when the labels on the input data are available. Several types of supervised algorithms exist, including linear methods, decision trees, neural networks, and support vector machines. Unsupervised learning is applied when labels are unknown for the input date. Clustering and principal component analysis (PCA) are frequently used unsupervised methods [88].

Nowadays, machine learning technology has been applied to almost every field of science and engineering on a global scale. Life science and healthcare have widely benefitted from machine learning and powerful algorithms are now available to diagnose disease, stratify patients, develop drugs, repurpose drugs, predict treatment outcomes, and recommend personalized treatment [89]. The past few years have seen an upsurge in the application of machine learning within microbiome research, following the publication of large accessible datasets such as The NIH Human Microbiome Project [90]. A variety of machine learning approaches, such as logistic regression, neural networks, and support vector machines, have been used to identify microbial features present in several disease states [91].

In a study by Zeevi et al., the authors successfully predicted post-meal glycemic response by training a regression model based on the individual’s microbiome features and personal information together with their diet’s nutrient profile [75]. A similar approach was employed in a later study to show that personalized glycemic response to different bread types can be predicted based on prior microbiome data [92]. In a recently published landmark study, authors reported a trained deep neural network model that could predict antibiotics based on structure. They applied the model on multiple chemical libraries and discovered a novel molecule with antibacterial effect [93].

The entire machine learning process is highly reliant on the quality of the input data and can be affected by factors including the implementation of the algorithms, definition of the parameters, and selection of the features. Projects using machine learning for microbiome studies and microbiome therapeutics will probably require information on microbiota, drugs, host metabolism and host-microbiota interactions. Table 3 provides a summary list of the representative databases with potential for application of the machine learning in microbiome field.

**Table 3:** List of the representative databases with potential for application of the machine learning in microbiome field

Database	Reference (URL)	Description
BacDive	https://bacdive.dsmz.de/	BacDive offers data on 81,827 bacterial and archaeal strains, including 14,091 type strains and thereby covers approx. 90% of the validly described species
Gold	https://gold.jgi.doe.gov/	Gold is a World Wide Web resource for comprehensive access to information regarding genome and metagenome sequencing projects, and their associated metadata
NCBI Microbial Genomes	https://www.ncbi.nlm.nih.gov/genome/microbes/	Microbial Genomes resource presents public data from prokaryotic genome sequencing projects
EnsemblBacteria	http://bacteria.ensembl.org/index.html	Ensembl Bacteria is a browser for bacterial and archaeal genomes
European Nucleotide Archive	https://www.ebi.ac.uk/ena/browser/home	The European Nucleotide Archive (ENA) provides a comprehensive record of the world’s nucleotide sequencing information, covering raw sequencing data, sequence assembly information and functional annotation
DrugBank	https://go.drugbank.com/	DrugBank, the world's most comprehensive and structured drug and molecular drug information resource
Super Natural	http://bioinf-applied.charite.de/supernatural_new/index.php?site=home	Super Natural II, a database of natural products. It contains 325,508 natural compounds (NCs), including information about the corresponding 2d structures, physicochemical properties, predicted toxicity class and potential vendors
ChEMBL	https://www.ebi.ac.uk/chembl/	ChEMBL is a manually curated database of bioactive molecules with drug-like properties
ChemSpider	http://www.chemspider.com/	ChemSpider is a free chemical structure database providing fast text and structure search access to over 100 million structures from hundreds of data sources
BindingDB	http://www.bindingdb.org/bind/index.jsp	BindingDB is a public, web-accessible database of measured binding affinities. BindingDB contains 41,328 Entries, each with a DOI, containing 2,259,122 binding data for 8,516 protein targets and 977,487 small molecules
MicrobiomeDB	https://microbiomedb.org/mbio/app/	A data-mining platform for interrogating microbiome experiments
UniProt	https://www.uniprot.org/	UniProt provides the scientific community with a comprehensive, high-quality and freely accessible resource of protein sequence and functional information
Virtual Metabolic Human	https://www.vmh.life/#home	The VMH database captures information on human and gut microbial metabolism and links this information to hundreds of diseases and nutritional data
Disbiome	https://disbiome.ugent.be/home	Disbiome® is a database covering microbial composition changes in different kinds of diseases, managed by Ghent University
eHOMD	http://www.homd.org/	eHOMD provides comprehensive curated information on the bacterial species present in the human aerodigestive tract (ADT), which encompasses the upper digestive and upper respiratory tracts, including the oral cavity, pharynx, nasal passages, sinuses and esophagus
HMDB	https://hmdb.ca/	The Human Metabolome Database (HMDB) is a freely available electronic database containing detailed information about small molecule metabolites found in the human body
MDB	https://db.cngb.org/microbiome/	Microbiome database involves the sequencing resource and metadata of ecological community samples of microorganisms, including both host-associated or environmental microbes
MGnify	https://www.ebi.ac.uk/metagenomics/	MGnify provides amplicon, assemblies,metabarcoding, metagenomes and metatranscriptomes data on human and environmental biomes
Human Microbiome Project	https://www.hmpdacc.org/	Genomic characterization of microbiota at five body sites (HMP1), and information on microbiota-human interactions in disease (iHMP)

## Perspective: Integration of Modeling and Machine Learning to design microbiome-aware personalized treatment

During the past decade, the gut microbiome has emerged as a biological system with high therapeutic potential, and advances in our understanding of the microbiome and its interaction with the host have opened a new horizon in biotechnology and precision medicine. There is strong evidence supporting the role of diet and microbiome in the triggering and progression of IBS, and targeting microbiota appears promising considering positive response of some patients to microbiome-related therapies. However, the complexity and heterogeneity of IBS and lack of highly predictive diagnostic and prognostic biomarkers resulted in unsatisfactory outcomes.

Progress in high-throughput technologies and bioinformatics has facilitated the acquisition of multi-dimensional clinical and biological data and the translation of these data into knowledge. Several studies have demonstrated the capacity to collect comprehensive, longitudinal datasets for individuals, including quantification of intestinal and dietary metabolite concentrations, classification and characterization of the host data (including diet, anthropometrics, lifestyle and disease background) and microbiome data (such as strain-level composition and abundance, metagenomics, meta-transcriptomics and metabolomics). However, most current studies involving interactions between human physiology, microbiome and food remain correlative rather than explanatory. A deeper understanding of the underlying mechanisms is important in designing safe and efficient novel therapeutic interventions, such as pre/probiotics, synbiotics, antibiotics and dietary regimes/food supplements.

Detecting the potentially interfering factors with efficacy of the synbiotics and dietary compounds and exploring underlying mechanisms, will require the development of algorithms that integrate multi-scale data and suggest the optimal combinations that would result in desired beneficial transformations. Recent studies tried to provide mechanistic insight by reconstructing genome-scale metabolic model of gut species and using these models to simulate host-microbiome-diet interactions [94, 95]. Despite their promise, limited coverage and low accuracy of the reconstructed metabolic models are major challenges for translation of these approaches. Moreover, there is currently no efficient approach to perform temporospatial simulation of species-level metabolic interactions.

**Fig. 4:**
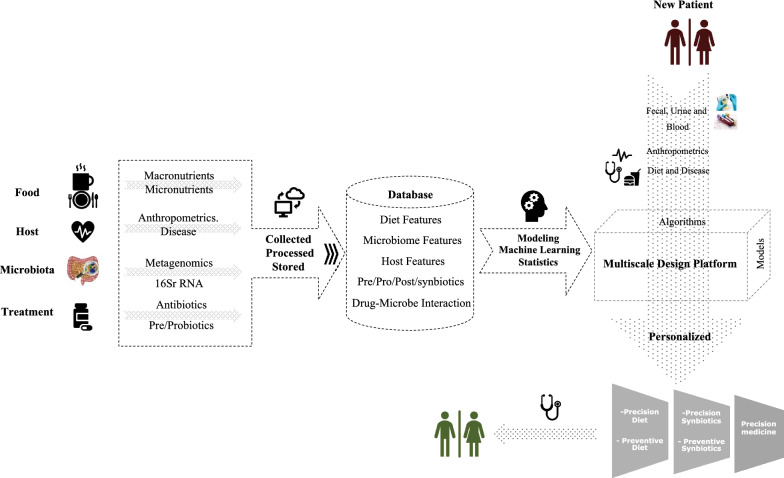
Microbiome-aware in silico platform. Schematic representation of the data driven platform that integrates multiscale modeling and artificial intelligence to provider deeper mechanistic understanding of microbiome and host response. This platform defines a dysbiosis fingerprint using person-specific data and employs algorithms to design precision diet/synbiotics to transfer this dysbiosis fingerprint towards symbiotic fingerprint. This platform can be used to formulate and to produce new generation of the optimally designed food supplements and pre/probiotics to improve desired trait for individuals or stratified populations

Ultimately, these advances will enable the development of in silico platforms that can integrate high dimensional data and provide mechanistic insight into host, microbiome, and diet interaction. Developed computational platform can integrate multi-dimensional datasets and provide a structured, curated and simulation ready database that allows for the implementation of the desired features, machine learning algorithms and predictive multiscale models (Fig. 4). Multiscale modeling can detect underlying mechanistic chain and causal mechanisms of disorders, complementing machine learning techniques that are agnostic to causality. Personalized models can be reconstructed based on measured variants for disease process in an individual patient and combined with machine learning, to create a personalized in silico pair of the physical condition. Developed platform can be employed for better characterization of the disorder and for identification of the potential therapies prior to clinical trials. Such in silico platforms have the potential to drive a paradigm shift in prevention, diagnosis, and treatment of the diseases in a personalize manner.

## Data Availability

There is no new data generated as part of this review.
